# Response to comment on: Toward an optimal contraception dosing strategy

**DOI:** 10.1371/journal.pcbi.1012649

**Published:** 2024-12-18

**Authors:** Brenda Lyn A. Gavina, Aurelio A. de los Reyes V, Mette S. Olufsen, Suzanne Lenhart, Johnny T. Ottesen

**Affiliations:** 1 Institute of Mathematics, University of the Philippines Diliman, Quezon City, Philippines; 2 Maritime Academy of Asia and the Pacific, Bataan, Philippines; 3 Biomedical Mathematics Group, Pioneer Research Center for Mathematical and Computational Sciences, Institute for Basic Science, Daejeon, Republic of Korea; 4 Department of Mathematics, North Carolina State University, Raleigh, North Carolina, United States of America; 5 Department of Mathematics, University of Tennessee, Knoxville, Tennessee, United States of America; 6 Department of Sciences, Roskilde University, Roskilde, Denmark; 7 Center for Mathematical Modeling—Human Health and Diseases, Roskilde University, Roskilde, Denmark; University of Michigan, UNITED STATES OF AMERICA

We appreciate the insightful comments on our recent paper, "Toward an optimal contraception dosing strategy." The detailed analysis and practical concerns raised regarding applying our findings in clinical setting are commendable.

From a clinical point of view, the concerns addressed in the Formal Comment, about applying theoretical models to real-world scenarios are entirely valid. However, as researchers, we know that any mathematical model inherently simplifies reality. Like any other field, our work serves as a step in a continuum of scientific inquiry—to gain quantitative insights into complex systems. The human body interacts with countless variables, many interconnected in ways that current science cannot fully quantify. As such, our study contributes to a broader understanding of contraception that may, over time, inform more personalized and effective treatments.

The observations phrased in (1), (2), and (3) about the inter- and intra-individual variability in patients is crucial. This variability makes it challenging to model the menstrual cycle precisely. However, this does not undermine the value of modeling studies; instead, it underscores the need for them. Modeling allows us to simulate different scenarios and explore the potential outcomes of various dosing strategies, helping narrow down the most promising approaches before testing these in clinical trials. For instance, we discussed how the optimal dosing profile adapts to a menstrual cycle of varied amplitude and length. In addition, we demonstrated the possibility of administration triggered by a specific biomarker, such as the E2 level. So even if some patients’ cycle length varies from month to month, their E2 level can be measured to know when to infuse a specific dose of contraceptive. This concept is analogous to studies where LH levels indicate the timing of administration in GnRH antagonist protocols [1,2].

Regarding (4), we recognize that the objectives of modeling studies are limited, just as those of clinical studies and experimental findings are. Our study considers the administration of exogenous hormones, and for simplicity, it does not account for pharmacokinetics. An obvious way to expand our findings is to couple the presented model with a PKPD model linking hormonal to a specific drug with known compounds and use parameter estimation to adapt the model to specific patients.

While such a study would be valuable it does not change our results showing how the exogenous hormone dose could be reduced if administered at the appropriate part of the cycle. Moreover, our study considers contraceptives containing estrogen and progesterone, such as implants and injections, and is not limited to pills alone. Some injections are administered monthly [3], implants deliver a continuous dose for up to three years [4], and while we agree that most pills are administered for a minimum of 21 days, there have been studies showing a low risk of ovulation for some women missing pill days [5], suggesting the possibility of new schedules and minimum effective doses that could further improve clinical practice.

In response to their concerns about estrogen-only preparations, we agree that such regimens pose significant risks, particularly in terms of endometrial health. The historical data referenced regarding the rise in endometrial cancer rates with certain cyclical combined contraceptives serves as a reminder of the importance of including progestin in contraceptive regimens. Our model does not advocate for the use of estrogen-only contraception without careful consideration of these risks. Instead, it suggests that there may be opportunities to optimize the timing and dosage of estrogen and progestin to reduce overall hormone exposure while maintaining efficacy and safety. Our study shows how a continuous infusion of exogenous hormone following a non-constant profile could significantly lower the drug dose. Furthermore, we have shown that the best dosing scheme involves estrogen and progesterone, which reduces the levels of both hormone components.

As they correctly pointed out in (5) and (6), the clinical acceptability of any contraceptive method depends not only on its efficacy in preventing ovulation but also on its ability to control the menstrual cycle and minimize side effects. The history of oral contraceptives has shown that even minor adjustments in hormone levels can have significant impacts on patient outcomes, both positive and negative. This is why any proposed dosing strategy is carefully tested in diverse populations before being recommended for widespread use.

We are pleased that our paper has sparked reflections on practical clinical issues, such as the variability in patients’ responses and the potential side effects of hormonal contraception. These are the considerations as we move from theoretical models to practical applications.
